# Contributions to the History of Development of the Teeth

**Published:** 1888-05

**Authors:** Carl Heitzmann, C. F. W. Bödecker


### Independent Practitioner.

VOL. IX. MAY, 1888. No. 5.

NOTE.—No paper published or to be published in another journal will be accepted for this department. All papers must be in the hands of the Editor before the first day of the month preceding that in which they are expected to appear. Extra copies will be furnished to each contributor of an accepted original article, and reprints, in pamphlet form, may be had at the cost of the paper, press-work and binding, if ordered when the manuscript is forwarded. The Editor and Publishers are not responsible for the opinions expressed by contributors. The journal is issued promptly, on the first day of each month.

### Original Communications.

### CONTRIBUTIONS TO THE HISTORY OF DEVELOPMENT OF THE TEETH.

BY CARL HEITZMANN, M. D., AND C. F. W. BÖDECKER, D. D. S., M. D. S.

CONTINUED FROM PAGE 175.

J. B. Gariot (Treatise on the Diseases of the Mouth. Am. Jour. Dent. Sc. Baltimore, 1843) described the follicles of the teeth as composed of a double fold which surrounds the germ of the tooth. The follicle resembles a serous membrane, and lines the interior of the socket to which it is attached. He further states that the follicle secretes an albuminous serum which lubricates the parts that it surrounds, and that the ossific matter is deposited upon the teeth by the vessels that penetrate the dental cavities.

M. Desirabode (Am. Jour. Dent. Science, 1847) concluded that the teeth are the products of a true secretive organ. They are developed in a fibrous sac which is derived from the fibrous tissues of the gums, and which is composed of a single and, not as others had stated, of a double membrane. This membrane (follicle) later becomes the alveolo-dental periosteum. Of the formation of the dentine Desirabode states that it is accomplished by secretion, and not by a direct calcification of the elements of the pulp. In regard to the development of the enamel, this author is of the opinion that it is secreted by the follicular membrane, as he observed enamel upon the teeth of those animals only in which he noticed that tissue. In conclusion, on the formation of the enamel, he says, "We content ourselves with the following one (fact), extracted and translated from a German anatomist (Valcherus Coiten) who wrote two hundred and fifty years ago; a period when anatomy was first brought to light." The teeth are formed from a species of mucus enclosed in the cavity of a follicle; they are developed like a rudimentary body, around which an incrustation is formed, differing in that respect from the formation of bone, which is effected through the medium of a cartilage.

Edward Lent (From a translation of The Am. Jour of Dent. Science, 1857, p. 562) is of the opinion that the nuclei of the odontoblasts elongate, form the dentinal canals (the dentinal fibers), and around them the basis-substance of the dentine is deposited by secretion. He observed that the cylindrical ivory cells (odontoblasts) are not present upon the surface of the whole pulp, but only upon those parts which precede the formation of the dentine. Lent further states that one odontoblast may lengthen and form an entire dentinal tube (fiber), but that it is possible for two or more to unite. In regard to the development of the enamel, this author gives two illustrations, but says nothing more about them than that they are specimens of enamel.

J. Foster Flagg (Dental Cosmos, Vol. XIV, pp. 580 and 633) is of opinion that a space exists between the enamel membrane and the dental pulp into which there is poured out a plasma derived from the dental pulp, which afterward forms the enamel rods. This author further states: "Between the enamel rods from the commencement of their formation to the completion of their proper length, we have a network of organic structure, through the instrumentality of which the complete hardness of the enamel is effected, and its future nourishment from the enamel membrane maintained. This final hardening takes place from the exterior inwardly, and thus it is that we find finished enamel hardest externally."

Henry Sewill (Dental Cosmos, Vol. XVII, p. 658) gives a good account of dental development, principally taken from the views of Marcusen, Dursy, Kölliker, Waldeyer, Robin, Magitot and Tomes.

Charles S. Tomes (A Manual of Dental Anatomy, Philadelphia, 1882), after detailed description of the several steps of the formation of the primitive fold into the jar-like enamel organ, and the origin of the stellate reticulum from the epithelia of the enamel organ, states that as soon as the dentinal papilla begins to develop, the ameloblasts become elongated and enlarged. He further says that the whole enamel organ is derived from the oral epithelium, and that all must be regarded as "epithelial structures." This author then describes the formation of the dentinal papilla and the dental sac, and returns to a more detailed description of the enamel organ, where he states "that it may fairly be concluded that the enamel cells as they are used up in the formation of enamel, are recruited from the cells of the stratum intermedium. In regard to the stellate reticulum, Tomes says that its functions and destination are not very clear. With reference to the external epithelium he says that it is of little interest, save that it is a matter of controversy what becomes of it. He also observed the bead-shaped varicosities in the enamel cord.

The cement, this author states, is formed by the alveolo-dental periosteum in the same manner as bone tissue, when present in thin layers. Of calcification of the enamel Tomes says: "I am distinctly of the opinion that the enamel is formed by the actual conversion of the cells of the enamel organ into enamel." He also noticed that the stellate tissue of the enamel organ disappears before the completion of the enamel, but does not state in what manner the enamel attains its normal thickness.

In regard to the calcification of the dentine, this author is of opinion that the odontoblasts, when the formation of dentine is most active, have a broad base, which is directed toward the dentinal cap. They are in close contact with one another as long as the formation of the dentine is active, but when this is completed the odontoblasts become more elongated and rounded. Tomes further says: "The dentine is, I believe, formed by the direct conversion of the odontoblast cells, just as is the enamel * * * and is derived from them, and from them alone. The three structures (the dentinal fibers, the dentinal sheath, and the matrix between the latter) may be taken as being three stages in the conversion of one and the same substance. The most external portions of the odontoblasts undergo a metamorphosis into a gelatinous matrix, which is the seat of calcification, while their most central portions remain soft and unaltered as the fibrils." In regard to the appearance of the globular territories, Tomes maintains the view that they are not formed by the odontoblasts, as he states "they are actually in the substance of the cap, their growth and coalescence obviously go on without any very immediate relation to the cells of the pulp. The occurrence of these globular forms, and consequent large interglobular spaces * * * is therefore an evidence of arrest of development rather than of any otherwise abnormal condition."

On the calcification of the cementum, Tomes says that the osteoblasts themselves become calcified, being derived from the inner wall of the dental follicle. He further endorses the statements made by his father, Sir John Tomes, and Mr. DeMorgan, which are given as follows: "Here (towards the bone) in the place of cells with elongated processes, or cells arranged in fiber-like lines, we find cells aggregated into a mass, and so closely packed as to leave little room for intermediate tissue. The cells appear to have increased in size at the cost of the processes which existed at an earlier stage, and formed a bond of union between them. Everywhere about growing bone a careful examination will reveal cells attached to its surface, while the surface of the bone itself will present a series of similar bodies ossified. To these we propose to give the name of osteal cells, as distinguished from lacunal and other cells." Tomes also describes the myxomatous reticulum of young pericementum, and then explains the development of cementum as follows: "The osteoblasts form both matrix and bone corpuscles; in Prof. Klein's words, 'each osteoblast by the peripheral portion of its cell substance gives origin to the osseous ground substance, while the central protoplasm around the nucleus persists with the latter as the nucleated bone cell. The bone cell and the space in which it lies become branched. For a row of osteoblasts we then find a row of oblong or round territories, each composed of matrix, and in it a nucleated branched cell. The outlines of individual territories are gradually lost, and we then have a continuous osseous lamina, with its bone cells. The ground substance is, from the outset, a network of fibrils; it is at first soft, but soon becomes impregnated with inorganic salts, the process commencing at the point of ossification.' The bone cells with their processes are situated in corresponding lacunæ and canaliculi, just as in the adult osseous substance."

R. Baume (Odontologische Forschungen, Leipzig, 1882) says that the dentine is formed by the membrana præformativa eboris (first described by Raschkow, 1835), which he suggests as existing between the newly formed dentine and the layer of odontoblasts, and which is composed of globules of different sizes. This author also states that this membrane cannot originate from ordinary connective tissue, but is specifically developed from the odontoblasts. He endorses the theory that both the dentine and enamel are formed by a secreting process. In regard to the external epithelium Baume says that it is lost long before the process of calcification of the enamel begins, but does not state what becomes of it.

With reference to the formation of the cementum, he believes that the tooth sac is the matrix for the cement. This author recognizes two distinct membranes as existing upon the outer surface of the enamel. One (the outer), a covering of cement substance (Nasmyth layer), the remains of the upper part of the tooth sac, and the other membrane which lies close upon the enamel prisms, the remains of the membrana præformativa (adamantina), a product of epithelial elements.

Concerning pigmentation of the enamel, this author explains that in such places there are inter-prismatic spaces present in the enamel that are filled *with air!*

R. R. Andrews (The New England Journal of Dentistry, Vol. II, p. 193, and Transactions of the Ninth International Medical Congress, 1887), speaking of the formation of the dentine, holds the opinion that the odontoblasts calcify and form the matrix (basis-substance) of the dentine, while the dentinal fibers are formed by separate pear-shaped cells situated between the odontoblasts. But the sheath of Neuman is formed by those parts of the odontoblasts which surround the dentinal fibers.

James E. Garretson (The New England Journal of Dentistry, 1883, Vol. II, p. 369), in an address before the New England Dental Society, said: "A mucous membrane does not dip down; it dips up. * * * Dentine, cementum and enamel are resultant of a common secretion, and this secretion lies with the dental pulp. There is no enamel pulp as propounded, and as is thought to be shown by the microscopists. * * * In the earliest days of fœtal existence, the jaws are planes of cartilage. These planes are overlaid by mucous membrane. Between the cartilage and the membrane the papillæ, known as dental germs, are first met with." * * * "A developing germ carries with it overlying mucous membrane, the membrane hugging it closely. This covering or envelope, constitutes a tunic;" * * * "A germ, originally microscopic, has enlarged until it stands in shape and size the representative of a tooth; this germ is enveloped in a double sac; it is overgrown on all its circumference by tissue which, later, is to express itself as alveolar process and gum." * * * "The formation of dentine completed, the covering of it with enamel begins; or rather this deposit is, to a degree, coincident with the dentinal formation. Secreted by the same pulp which forms the dentine, the same secretion, some portion finds its way into and through the primary sac. As it passes through this sac to be moulded against the second, it is modified by the epithelial surface which constitutes the outer face of the tunica propria." * * * "Between the enamel, thus formed, and the dentine, exists the primary sac; simply the modified mucous membrane, which we first saw overlying the papilla. The sac of mucous membrane continues to exist between these two hard bodies, and receives and modifies, for the support of the enamel, the liquor sanguinis found in the dentinal tubercles and intertubular substance. This tunica propria is the enamel membrane. It is from this that we receive impressions of pain when it becomes exposed by a break in the continuity of enamel." * * * "Enamel has no special pulp, as propounded by the histologists. It is also understood that it calcifies from the outside inward, and *not* from the inside outward."

(TO BE CONTINUED.)

